# “Precious beyond measure”: rethinking the current approach to diversity

**DOI:** 10.3389/fpsyg.2023.1336590

**Published:** 2023-12-08

**Authors:** Samantha R. Mattheiss

**Affiliations:** School of Arts and Sciences, Felician University, Lodi, NJ, United States

**Keywords:** diversity, discrimination, group, identity, beauty, growth, unity

## Introduction

In recent years, there has been great effort to establish new, and reinforce existing, groups with the goal of capturing the wide range of phenotypes represented in the human population. Such groups, or identities, include neurodiverse, straight, aromantic, nonbinary, white, black, interracial, Hispanic or LatinX, and non-Hispanic or LatinX. With an emphasis on diversity, social movements have emerged which rightly aim to support marginalized groups, eradicate discrimination, and foster improvement amongst countless lives. Such movements align with psychological research demonstrating that group identification can protect against depression (Cruwys et al., 2014) and promote well-being by fostering a sense of belonging and meaning (Jetten et al., 2017; Haslam et al., 2018). Despite these advances, hate crimes have risen in recent years (Miller and Rivas, 2022; Farrell and Lockwood, 2023) and global conflict persists (Pandey et al., 2023; United Nations, 2023). While group identification is inarguably beneficial, does overemphasizing categories come with a cost? Moreover, is there evidence pointing toward another way to effectively embrace the beautiful, infinite diversity of humanity?

## Continuum

To begin with, many of the characteristics on which we tend to categorize ourselves are, in fact, not categorical in nature, but rather are inherently continuous—in that each facet is better represented on a continuum, or spectrum. In terms of gender, for example, research shows that each individual shares some characteristics historically thought to be “male” and others “female,” regardless of biological sex (Monro, 2005). The same is true for sexuality (Savin-Williams, 2014), with many individuals across the sexual identity spectrum reporting multiple identities as well as changes in identities over time (Ruberg and Ruelos, 2020).

Moreover, it is increasingly clear that cognitive and emotional traits lie on a continuum, rather than in discrete categories. For example, not all individuals within clinically defined diagnostic categories, such as depression, benefit from the same treatment, suggesting that existing clinical categories may not accurately represent phenotypes. In fact, van Os et al. (2023) demonstrated that a transdiagnostic approach, in which a number of factors (e.g., symptom dimensions such as persistence; clinical factors such as early adversity) are taken into account, is more valuable to patients than the existing categorical approach (van Os et al., 2023). Neurobiological (Gray et al., 2020; Dugré et al., 2022; Feng et al., 2022; Li et al., 2022) and genetic (Hoy et al., 2022; Chawner et al., 2023; David et al., 2023) evidence also supports the heterogeneity of symptoms within neuropsychiatric disorders, with biomarkers predicting transdiagnostic symptoms rather than discrete diagnoses.

Other research suggests that a categorical approach may also be inadequate for capturing racial and ethnic identities. For example, an increasing multiracial and multiethnic—also termed mixed (Törngren et al., 2021)—population (Livingston, 2020) challenges the rigidity of existing racial and ethnic identities (DaCosta, 2020). Further, with genetic ancestry testing (e.g., 23&Me, Inc.), some receive information about their ancestry that contradicts their existing, socially constructed racial and ethnic identities (Elliott and Brodwin, 2002; Shim et al., 2018), thereby potentially impacting one's group identity (Theunissen, 2022). Together, such findings suggest that a rigid approach to racial and ethnic identities is insufficient.

## Beyond measure

Such findings across gender, sex, ability, disability, disorder, race, and ethnicity suggest that diversity may be beyond what human language can capture. Indeed, each person differs on countless characteristics, inspired by a unique set of over 100,000 (and counting; Salzberg, 2018) human genes, over 100 billion neurons (Toga et al., 2012), and over 700 trillion connections between neurons (The Brain Preservation Foundation, 2015). Each person differs in their life experiences–beginning prenatally–with exposure to distinct levels of nutrients, hormones, and toxins prior to birth (e.g., Ferreira, 1965; Maccari et al., 2003; Kingston and Tough, 2014; Gómez-Roig et al., 2021); and then, different experiences during birth, infancy, childhood, and beyond (e.g., Cannon et al., 2002; Bouchard and McGue, 2003; Annear et al., 2014; Hughes et al., 2017). As such, our differences are inherently beyond what we can categorize with language.

## Costs associated with group identification

In addition to the challenge of maintaining categories to represent the true diversity of humanity, there are also risks associated with increasing the saliency of groups. For example, experimentally manipulated categories resulted in increased perceptions of differences between the “ingroup”—the group with which an individual identifies—and “outgroup” (Tajfel et al., 1971); as well as increased discrimination of “outgroup” members (Turner et al., 1979). Moreover, once individuals identify with a group, their self-concept may be influenced by perceptions of their group (Turner et al., 1987; Hogg and Williams, 2000), even when those perceptions are inaccurate, negative, or inconsistent with their personality identity (Hogg and Williams, 2000; Sim et al., 2014).

## An alternative approach

### Appreciation of beauty

There are potential benefits and costs to group identification. As such, I am not proposing that we get rid of categories or shift any group movements aside, but rather that we take a moment to pause, and recognize the incredible and beautiful diversity of the human person—one which is simply beyond categories.

Cognitive neuroscientist David Eaglemen describes a sense of awe at the human mind, which, he describes is “inimitable, mysterious…precious beyond measure” and “more remarkable than any orb in the sky” (Eagleman and Downer, 2016).

What if we were to emphasize the preciousness of each person we encounter? What if we were to actively seek beauty and goodness in the individual next to us, across from us, ahead of us, behind us? In fact, experiences of beauty are related to self-transcendence (Diessner et al., 2018); awe (Keltner and Haidt, 2003); prosocial behavior (Zhang et al., 2014); a reduction in the self-other distinction (D'Aquili and Newberg, 2000); decreased discrimination (for review, see Pohling and Diessner, 2016); and greater love of humanity (for review, see Pohling and Diessner, 2016).

In addition to appreciation of the beauty of the individual human, an appreciation of the beauty of unity-in-diversity (Diessner, 2004), or when “various diverse elements that are integrated into unities” (Diessner et al., 2006, p. 307), is also beneficial. Specifically, appreciating the beauty of unity-in-diversity lends to effective moral reasoning about valuation of individual differences amongst unity; greater acceptance of differences; and decreased discrimination (Diessner et al., 2006; Diessner and Niemiec, 2023).

Given such findings, it just might be in recognizing the beauty of each individual- and of the diversity of humanity- that we become increasingly united in our common humanity.

### Recategorization

In addition to emphasizing the beauty of each immeasurably unique human person, might we also emphasize our similarities? Although we are incredibly unique, we also share 99.9% of genes (Nhgri, 2019). Moreover, populations across the globe share a common earth; some share a common workforce, neighborhood, or school; and countless share inter- and intra-personal joys and struggles. Across cultures, individuals share in the expression of the universal emotions (Russell, 1994), as well as the developmental trajectories designated by the human genome (Venter et al., 2001).

Appreciating similarities may be beneficial. In fact, feeling similar to someone is not only a core component of social connection (Seppala et al., 2013), but also increases feelings of connectedness which extend to other members of the “outgroup” (Galinsky et al., 2005). Social connection is linked to other benefits as well, including improved health, wellbeing, and even longevity (Seppala et al., 2013).

Several studies have empirically examined the effects of recategorization of groups based on similarities. For example, evidence supports the common ingroup identity model (Gaertner et al., 1989, 1993), which suggests that if individuals of two separate groups reorganize their identities such that they belong to a single, inclusive group, they will experience more positive attitudes toward each other (Gaertner et al., 1993). Studies demonstrate that even experiencing a shared high stress activity (vs. low stress and vs. unshared/different activities) can foster prosocial behavior (Dovidio and Morris, 1975); and that reorganization of one's identity group can foster positive attitudes toward out-group members (Gaertner et al., 1989).

In another study, recategorizing a partner in black – white friendship dyads as belonging to the same subordinate group as oneself led to greater concern for others as well as security in the relationship (Lemay and Ryan, 2021). Interestingly, perceived similarity was responsible for the effect. Moreover, both motivation and relationship security predicted prosocial behavior and relationship satisfaction.

In a similar line of research, shared sufferings, including perceptions of hate and prejudice, have been shown to foster group identity and elicit within-group oneness (Whitehouse et al., 2017; Walters et al., 2020). These findings deepen our understanding of intergroup conflict, yet they may also be relevant here. Across the globe, we share sufferings such as losing a loved one and enduring physical illness. It's possible that recognizing shared sufferings can lead to benefits associated with perceived similarity.

Overall, such findings imply that an emphasis on identifying similarities between self and other—even amidst an apparent difference in group identity—may be key.

## Translation into practice

The translation of such findings into real-world practices presents a challenge, yet it seems surmountable. First, it is generally accepted that individual traits, including personality and cognitive traits, change with experience (e.g., Peterson and Seligman, 2004; Cloninger et al., 2019; Zwir et al., 2019; De Vries et al., 2021). Moreover, the biopsychosocial basis of personality demonstrates the drive to align one's actions with their goals and values (Garcia et al., 2023), with more alignment corresponding to greater implementation of virtue in action (Moreira et al., 2022). Together, such findings alongside neurobiological mechanisms of change (i.e., brain plasticity, Kolb and Whishaw, 1998), suggest that positive intrapersonal and thereby, interpersonal, change is possible.

Additionally, several studies demonstrate the efficacy of practices that enhance intra- and interpersonal growth. These include role-taking and social play amongst children (e.g., Ahammer and Murray, 1979), mobile phone applications (e.g., Morris et al., 2010), focus groups (e.g., (Lundqvist et al., 2010), therapy (Bamelis et al., 2014) including a person-centered approach (Garcia et al., 2023), interventions (e.g., Shonin et al., 2015), social emotional learning (SEL) curricula (Duchesneau, 2020; Murano et al., 2020), and public awareness campaigns (e.g., Agha, 2003). Such practices have been shown to be effective across populations, including those diagnosed with traumatic brain injury (Lundqvist et al., 2010) and personality disorders (Bamelis et al., 2014).

Translation of the proposed approach into real-world practices might begin with the modalities described above. Future research can develop and test the effectiveness of specific practices.

It's also important to note that many existing practices incorporate other approaches to reduce discrimination (e.g., Devine et al., 2012; Gronholm et al., 2017; Windisch et al., 2021). The proposed approach here does not negate existing approaches, but rather aims to add a new perspective to the discussion.

## Striving toward equality

Finally, this approach requires a concomitant striving toward equality and respect for all persons. Intergroup inequality not only drives group identity and behavior (Lei and Vesely, 2010), but also signifies deep injustice. The proposed approach to diversity calls for equity as a starting point. Yet the relationship is bidirectional. If the approach fosters positive personality growth, prosocial and altruistic action should ensue (see Garcia et al., 2023), which should reduce inequality. Moreover, emphasizing our common humanity may, in turn, promote a greater desire for and action toward equality (see Tropp and Barlow, 2018).

## Conclusion

Overall, existing evidence challenges the current approach to diversity. Such points to another all-encompassing approach to diversity. This approach fosters an appreciation of the beauty of each individual, exquisitely unique beyond what human language can ultimately communicate. It also promotes a search for similarities between oneself and the other, and possibly a reorganization of transient, subordinate groups based on commonalities. With this approach, we celebrate the splendorous differences across the human population while also recognizing our common humanity. With an awareness of the immeasurable diversity of humanity, we just might find another beauty, described by Diessner et al. (2006)—one which is elicited by the coexistence of each and every one of us unrepeatable and exquisitely unique.

## Author contributions

SM: Conceptualization, Writing – original draft, Writing – review & editing.

## References

[B1] Agha S. (2003). The impact of a mass media campaign on personal risk perception, perceived self-efficacy and on other behavioural predictors. AIDS Care. 15, 749–762. 10.1080/09540120310001618603 14617497

[B2] Ahammer I. M. Murray J. P. (1979). Kindness in the kindergarten: The relative influence of role playing and prosocial television in facilitating altruism. Int. J. Behav. Dev. 2, 133–157. 10.1177/016502547900200203

[B3] Annear M. Keeling S. Wilkinson T. I. M. Cushman G. Gidlow B. O. B. Hopkins H. (2014). Environmental influences on healthy and active ageing: a systematic review. Aging Soc. 34, 590–622. 10.1017/S0144686X1200116X

[B4] Bamelis L. L. M. Evers S. M. A. A. Spinhoven P. Arntz A. (2014). Results of a multicenter randomized controlled trial of the clinical effectiveness of schema therapy for personality disorders. Am. J. Psychiatr. 171, 305–322. 10.1176/appi.ajp.2013.12040518 24322378

[B5] Bouchard T. J. Jr. McGue M. (2003). Genetic and environmental influences on human psychological differences. J. Neurobiol. 54, 4–45. 10.1002/neu.10160 12486697

[B6] Cannon M. Jones P. B. Murray R. M. (2002). Obstetric complications and schizophrenia: historical and meta-analytic review. Am. J. Psychiatry 159, 1080–1092. 10.1176/appi.ajp.159.7.1080 12091183

[B7] Chawner S. J. R. A. Evans A. Williams I. I. Owen N. Hall M. J. J. (2023). Sleep disturbance as a transdiagnostic marker of psychiatric risk in children with neurodevelopmental risk genetic conditions. Transl. Psychiatr. 13, 7. 10.1038/s41398-022-02296-z 36631438 PMC9834234

[B8] Cloninger C. R. Cloninger K. M. Zwir I. Keltikangas-Järvinen L. (2019). (2019). The complex genetics and biology of human temperament: a review of traditional concepts in relation to new molecular findings. Transl. Psychiatr. 9, 1–21. 10.1038/s41398-019-0621-4 31712636 PMC6848211

[B9] Cruwys T. Haslam S. A. Dingle G. A. Haslam C. Jetten J. (2014). Depression and social identity: an integrative review. Pers. Soc. Psychol. Review 18, 215–238. 10.1177/1088868314523839 24727974

[B10] DaCosta K. A. (2020). Multiracial categorization, identity, and policy in (mixed) racial formations. Ann. Rev. Sociol. 46, 335–353. 10.1146/annurev-soc-121919-054649

[B11] D'Aquili E. Newberg A. (2000). The Neuropsychology of Aesthetic Spirituala and Mystical States. Middlefield, CT: Zygon.

[B12] David F. S. Stein F. Andlauer T. F. M. Streit F. Witt S. H. Herms S.. (2023). Genetic contributions to transdiagnostic symptom dimensions in patients with major depressive disorder, bipolar disorder, and schizophrenia spectrum disorders. Schizophrenia Res. 252, 161–171. 10.1016/j.schres.2023.01.002 36652833

[B13] De Vries D. Spengler J. H. Frintrup M. A. Mussel P. (2021). Personality development in emerging adulthood—how the perception of life events and mindset affect personality trait change. Fronti. Psychol. 12, 671421. 10.3389/fpsyg.2021.671421 34234715 PMC8256263

[B14] Devine P. G. Forscher P. S. Austin A. J. Cox W. T. L. (2012). Long-term reduction in implicit race bias: a prejudice habit-breaking intervention. J. Exp. Soc. Psychol. 48, 1267. 10.1016/j.jesp.2012.06.003 23524616 PMC3603687

[B15] Diessner R. (2004). “Beauty and moral education, in: Center for Global Integrated Education Committee,” in Proceedings of CGIE's 2004 International Forum on Integrated Education and Educational Reform. Claremont, CA: Center for Global Integrated Education, 27–47.

[B16] Diessner R. Niemiec R. M. (2023). Can beauty save the world? Appreciation of beauty predicts proenvironmental behavior and moral elevation better than 23 other character strengths. Ecopsychology 15, 93–109. 10.1089/eco.2022.0047

[B17] Diessner R. Pohling R. Stacy S. Güsewell A. (2018). Trait appreciation of beauty: a story of love, transcendence, and inquiry. Rev. Gen. Psychol. 22, 377–397. 10.1037/gpr0000166

[B18] Diessner R. Rust T. Solom R. C. Frost N. Parsons L. (2006). Beauty and hope: a moral beauty intervention. J. Moral Educ. 35, 301–317. 10.1080/03057240600874430

[B19] Dovidio J. F. Morris W. N. (1975). Effects of stress and commonality of fate on helping behavior. J. Pers. Soc. Psychol. 31, 145. 10.1037/h0076236 1117404

[B20] Duchesneau N. (2020). Social, Emotional, and Academic Development Through an Equity Lens. Washington, DC: Education Trust.

[B21] Dugré J. R. Eickhoff S. B. Potvin S. (2022). Meta-analytical transdiagnostic neural correlates in common pediatric psychiatric disorders. Sci. Rep. 12, 1–12. 10.1038/s41598-022-08909-3 35318371 PMC8941086

[B22] Eagleman D. Downer J. (2016). Brain and Behavior: A Cognitive Neuroscience Perspective. Oxford: Oxford University Press.

[B23] Elliott C. Brodwin P. (2002). Identity and genetic ancestry tracing. BMJ 325, 1469. 10.1136/bmj.325.7378.1469 12493671 PMC139044

[B24] Farrell A. Lockwood S. (2023). Addressing hate crime in the 21st Century: trends, threats, and opportunities for intervention. Ann. Rev. Criminol. 6, 107–130. 10.1146/annurev-criminol-030920-091908

[B25] Feng C. Huang W. Xu K. Stewart J. L. Camilleri J. A. Yang X.. (2022). Neural substrates of motivational dysfunction across neuropsychiatric conditions: evidence from meta-analysis and lesion network mapping. Clin. Psychol. Rev. 96, 102189. 10.1016/j.cpr.2022.102189 35908312 PMC9720091

[B26] Ferreira A. J. (1965). Emotional factors in prenatal environment a Review. The J. Nerv. Mental Dis. 141, 108–118. 10.1097/00005053-196507000-00011 5320604

[B27] Gaertner S. L. Dovidio J. F. Anastasio P. A. Bachman B. A. Rust M. C. (1993). The common ingroup identity model: recategorization and the reduction of intergroup bias. Eur. Rev. Soc. Psychol. 4, 1–26. 10.1080/14792779343000004

[B28] Gaertner S. L. Mann J. Murrell A. Dovidio J. F. (1989). Reducing intergroup bias: the benefits of recategorization. J. Pers. Soc. Psychol. 57, 239. 10.1037/0022-3514.57.2.239

[B29] Galinsky A. D. Ku G. Wang C. S. (2005). Perspective-taking and self-other overlap: fostering social bonds and facilitating social coordination. Group Proc. Intergr. Relat. 8, 109–124. 10.1177/1368430205051060

[B30] Garcia D. Cloninger K. M. Cloninger C. R. (2023). Coherence of character and temperament drives personality change toward well being in person-centered therapy. Curr. Opin. Psychiatr. 36, 60. 10.1097/YCO.0000000000000833 36449732 PMC9794122

[B31] Gómez-Roig M. D. Pascal R. Cahuana M. J. García-Algar O. Sebastiani G. Andreu-Fernández V.. (2021). Environmental exposure during pregnancy: influence on prenatal development and early life: a comprehensive review. Fetal Diag. Ther. 48, 245–257. 10.1159/000514884 33735860

[B32] Gray J. P. Müller V. I. Eickhoff S. B. Fox P. T. (2020). Multimodal abnormalities of brain structure and function in major depressive disorder: a meta-analysis of neuroimaging studies. The Am. J. Psychiatr. 177, 422. 10.1176/appi.ajp.2019.19050560 32098488 PMC7294300

[B33] Gronholm P. C. Henderson C. Deb T. Thornicroft G. (2017). Interventions to reduce discrimination and stigma: the state of the art. Soc. Psychiatr. Psychiatr. Epidemiol. 52, 249–258. 10.1007/s00127-017-1341-9 28144713 PMC5344948

[B34] Haslam C. Jetten J. Cruwys T. Dingle G. Haslam S. A. (2018). The New Psychology of Health: Unlocking the Social Cure. London: Routledge.

[B35] Hogg M. A. Williams K. D. (2000). From I to we: social identity and the collective self. Group Dy. Theor. Res. Prac. 4, 81. 10.1037/1089-2699.4.1.81

[B36] Hoy N. Lynch S. Waszczuk M. Reppermund S. Mewton L. (2022). Investigating the molecular genetic, genomic, brain structural, and brain functional correlates of latent transdiagnostic dimensions of psychopathology across the lifespan: protocol for a systematic review and meta-analysis of cross-sectional and longitudinal studies in the general population. Front. Psychiatr. 13, 1036794. 10.3389/fpsyt.2022.1036794 PMC9669375 36405912

[B37] Hughes K. Bellis M. A. Hardcastle K. A. Sethi D. Butchart A. Mikton C.. (2017). The effect of multiple adverse childhood experiences on health: a systematic review and meta-analysis. Lancet Public Health 2, e356–66. 10.1016/S2468-2667(17)30118-4 29253477

[B38] Jetten J. Haslam S. A. Cruwys T. Greenaway K. H. Haslam C. Steffens N. K.. (2017). Advancing the social identity approach to health and well-being: Progressing the social cure research agenda. Eur. J. Soc. Psychol. 47, 789–802. 10.1002/ejsp.2333

[B39] Keltner D. Haidt J. (2003). Approaching awe, a moral, spiritual, and aesthetic emotion. Cognit. Emot. 17, 297–314. 10.1080/02699930302297 29715721

[B40] Kingston D. Tough S. (2014). Prenatal and postnatal maternal mental health and school-age child development: a systematic review. Mat. Child Health J. 18, 1728–1741. 10.1007/s10995-013-1418-3 24352625

[B41] Kolb B. Whishaw I. Q. (1998). Brain plasticity and behavior. Annu. Rev. Psychol 49, 43–64. 10.1146/annurev.psych.49.1.43 9496621

[B42] Lei V. Vesely F. (2010). In-group versus out-group trust: the impact of income inequality. Southern Econ. J. 76, 1049–1063. 10.4284/sej.2010.76.4.1049

[B43] Lemay E. P. Ryan J. E. (2021). Common ingroup identity, perceived similarity, and communal interracial relationships. Pers. Soc. Psychol. Bullet. 47, 985–1003. 10.1177/0146167220953984 32886043

[B44] Li M. Dahmani L. Hubbard C. S. Hu Y. Wang M. Wang D.. (2022). Individualized functional connectome identified generalizable biomarkers for psychiatric symptoms in transdiagnostic patients. Neuropsychopharmacology 48, 633–641. 10.1038/s41386-022-01500-4 36402836 PMC9938230

[B45] Livingston G. (2020). The Rise of Multiracial and Multiethnic Babies in the U.S. Available online at: https://www.pewresearch.org/fact-tank/2017/06/06/the-rise-of-multiracial-and-multiethnic-babies-in-the-u-s/#:~:text=One%2Din%2Dseven%20U.S.%20infants,Virginia%20legalized%20interracial%20marriage (accessed August 12, 2023).

[B46] Lundqvist A. Linnros H. Orlenius H. Samuelsson K. (2010). Improved self-awareness and coping strategies for patients with acquired brain injury—A group therapy programme. Brain Injury 24, 823–832. 10.3109/02699051003724986 20377345

[B47] Maccari S. Darnaudery M. Morley-Fletcher S. Zuena A. R. Cinque C. Van Reeth O.. (2003). Prenatal stress and long-term consequences: implications of glucocorticoid hormones. Neurosci. Biobehav. Rev. 27, 119–127. 10.1016/S0149-7634(03)00014-9 12732228

[B48] Miller C. Rivas R. C. (2022). The Year in Hate and Extremism Report 2021. Montgomery, AL: Southern Poverty Law Center.

[B49] Monro S. (2005). Beyond male and female: poststructuralism and the spectrum of gender. Int. J. Transgenderism 8, 3–22. 10.1300/J485v08n01_02

[B50] Moreira P. A. S. Inman R. A. Cloninger C. R. (2022). Virtues in action are related to the integration of both temperament and character: Comparing the VIA classification of virtues and Cloninger's biopsychosocial model of personality. J. Positive Psychol. 17, 858–875. 10.1080/17439760.2021.1975158

[B51] Morris M. E. Kathawala Q. Leen T. K. Gorenstein E. E. Guilak F. Labhard M.. (2010). Mobile therapy: case study evaluations of a cell phone application for emotional self-awareness. J. Med. Int. Res. 12, e1371. 10.2196/jmir.1371 20439251 PMC2885784

[B52] Murano D. Sawyer J. E. Lipnevich A. A. (2020). A meta-analytic review of preschool social and emotional learning interventions. Rev. Educ. Res. Month 21, 1–37. 10.3102/0034654320914743

[B53] Nhgri (2019). Genetics vs. Genomics Fact Sheet. Available online at: https://www.genome.gov/about-genomics/fact-sheets/Genetics-vs-Genomics#:~:text=All%20human%20beings%20are%2099,~9.about%20the%20causes%20of%20diseases (accessed August 6, 2023).

[B54] Pandey D. K. Lucey B. M. Kumar S. (2023). Border disputes, conflicts, war, and financial markets research: a systematic review. Res. Int. Bus. Finance 101972. 10.1016/j.ribaf.2023.101972

[B55] Peterson C. Seligman M. E. P. (2004). Character Strengths and Virtues: A Handbook and Classification. Oxford: Oxford University Press.

[B56] Pohling R. Diessner R. (2016). Moral elevation and moral beauty: a review of the empirical literature. Rev. Gen. Psychol. 20, 412–425. 10.1037/gpr0000089

[B57] Ruberg B. Ruelos S. (2020). Data for queer lives: how LGBTQ gender and sexuality identities challenge norms of demographics. Big Data Soc. 7, 2053951720933286. 10.1177/2053951720933286

[B58] Russell J. A. (1994). Is there universal recognition of emotion from facial expression? A review of the cross-cultural studies. Psychol. Bullet. 115, 102. 10.1037/0033-2909.115.1.102 8202574

[B59] Salzberg S. L. (2018). Open questions: How many genes do we have? BMC Biol. 16, 94. 10.1186/s12915-018-0564-x 30124169 PMC6100717

[B60] Savin-Williams R. C. (2014). An exploratory study of the categorical versus spectrum nature of sexual orientation. The J. Sex Res. 51, 446–453. 10.1080/00224499.2013.871691 24559054

[B61] Seppala E. Rossomando T. Doty J. R. (2013). Social connection and compassion: important predictors of health and well-being. Soc. Res. Int. Q. 80, 45. 10.1353/sor.2013.0027

[B62] Shim J. K. Rab Alam S. Aouizerat B. E. (2018). Knowing something versus feeling different: the effects and non-effects of genetic ancestry on racial identity. New Genetics Soc. Critic. Stu. Contemp. Biosci. 37, 44–66. 10.1080/14636778.2018.1430560 31666800 PMC6820700

[B63] Shonin E. Van Gordon W. Compare A. Zangeneh M. Griffiths M. D. (2015). Buddhist-derived loving-kindness and compassion meditation for the treatment of psychopathology: a systematic review. Mindfulness 6, 1161–1180. 10.1007/s12671-014-0368-1

[B64] Sim J. J. Goyle A. McKedy W. Eidelman S. Correll J. (2014). How social identity shapes the working self-concept. J. Exp. Soc. Psychol. 55, 271–277. 10.1016/j.jesp.2014.07.015

[B65] Tajfel H. Billig M. G. Bundy R. P. Flament C. (1971). Social categorization and intergroup behaviour. Eur. J. Soc. Psychol. 1, 149–178. 10.1002/ejsp.2420010202

[B66] The Brain Preservation Foundation (2015). Connectome – The Brain Preservation Foundation. Available online at: https://www.brainpreservation.org/content-2/connectome/ (accessed August 6, 2023).

[B67] Theunissen C. A. (2022). The effects of DNA test results on biological and family identities. Genealogy 6, 17. 10.3390/genealogy6010017

[B68] Toga A. W. Clark K. A. Thompson P. M. Shattuck D. W. Van Horn J. D. (2012). Mapping the human connectome. Neurosurgery 71, 1–5. 10.1227/NEU.0b013e318258e9ff 22705717 PMC3555558

[B69] Törngren S. O. Irastorza N. Rodríguez-García D. (2021). Understanding multiethnic and multiracial experiences globally: towards a conceptual framework of mixedness. J. Ethnic Migration Stu. 47, 763–781. 10.1080/1369183X.2019.1654150

[B70] Tropp L. R. Barlow F. K. (2018). Making advantaged racial groups care about inequality: intergroup contact as a route to psychological investment. Curr. Directions Psychol. Sci. 27, 194–199. 10.1177/0963721417743282

[B71] Turner J. C. Brown R. J. Tajfel H. (1979). Social comparison and group interest in ingroup favouritism. Eur. J. Soc. Psychol. 9, 187–204. 10.1002/ejsp.2420090207

[B72] Turner J. C. Hogg M. A. Oakes P. J. Reicher S. D. Wetherell M. S. (1987). Rediscovering the Social Group: A Self-Categorization Theory. London: Basil Blackwell.

[B73] United Nations (2023). A New Era of Conflict and Violence. United Nations, UN75 2020 and Beyond. Available online at: https://www.un.org/en/un75/new-era-conflict-and-violence (accessed November 9, 2023).

[B74] van Os J. Pries L. K. Ten Have M. van Dorsselaer R. Bak S. Guloksuz S.. (2023). Context v. algorithm: evidence that a transdiagnostic framework of contextual clinical characterization is of more clinical value than categorical diagnosis. Psychol. Med. 53, 1825–1833. 10.1017/S0033291721003445 37310330 PMC10106290

[B75] Venter J. C. Adams M. D. Myers E. W. Li P. W. Mural R. J. Sutton G. G.. (2001). The sequence of the human genome. Science 291, 1304–1351. 10.1126/science.1058040 11181995

[B76] Walters M. A. Paterson J. L. McDonnell L. Brown R. (2020). Group identity, empathy and shared suffering: Understanding the ‘community' impacts of anti-LGBT and Islamophobic hate crimes. Int. Rev. Victimol. 26, 143–162. 10.1177/0269758019833284

[B77] Whitehouse H. Jong J. Buhrmester M. D. Gómez Á. Bastian B. Kavanagh C. M.. (2017). The evolution of extreme cooperation via shared dysphoric experiences. Sci. Rep. 7, 1–10. 10.1038/srep44292 28290499 PMC5349572

[B78] Windisch S. Wiedlitzka S. Olaghere A. (2021). PROTOCOL: Online interventions for reducing hate speech and cyberhate: a systematic review. Campbell Syst. Rev. 17, 1133. 10.1002/cl2.1133 PMC8356355 37050974

[B79] Zhang J. W. Piff P. K. Iyer R. Koleva S. Keltner D. (2014). An occasion for unselfing: beautiful nature leads to prosociality. J. Environ. Psychol. 37, 61–72. 10.1016/j.jenvp.2013.11.008

[B80] Zwir I. Del-Val C. Arnedo J. Pulkki-Råback L. Konte B. Yang S. S.. (2019). Three genetic–environmental networks for human personality. Mol. Psychiatr. 26, 3858–3875. 10.1038/s41380-019-0579-x PMC8550959 31748689

